# Improving the Properties of Laser-Welded Al–Zn–Mg–Cu Alloy Joints by Aging and Double-Sided Ultrasonic Impact Compound Treatment

**DOI:** 10.3390/ma14112742

**Published:** 2021-05-22

**Authors:** Furong Chen, Chenghao Liu

**Affiliations:** School of Materials Science and Engineering, Inner Mongolia University of Technology, Hohhot 010051, China; cfr7075@imut.edu.cn

**Keywords:** 7075 aluminum alloy, welded joint, aging, ultrasonic impact treatment, microstructure, EBSD, mechanical properties

## Abstract

To improve the loose structure and serious porosity of (Al–Zn–Mg–Cu) 7075 aluminum alloy laser-welded joints, aging treatment, double-sided ultrasonic impact treatment (DSUIT), and a combination of aging and DSUIT (A–DSUIT) were used to treat joints. In this experiment, the mechanism of A–DSUIT on the microstructure and properties of welded joints was analyzed. The microstructure of the welded joints was observed using optical microscopy, scanning electron microscopy, and electron backscatter diffraction (EBSD). The hardness and tensile properties of the welded components under the different processes were examined via Vickers hardness test and a universal tensile testing machine. The results showed that, after the aging treatment, the dendritic structure of the welded joints transformed into an equiaxed crystal structure. Moreover, the residual tensile stress generated in the welding process was weakened, and the hardness and tensile strength were significantly improved. After DSUIT, a plastic deformation layer of a certain thickness was generated from the surface downward, and the residual compressive stress was introduced to a certain depth of the joint. However, the weld zone unaffected by DSUIT still exhibited residual tensile stress. The inner microhardness of the joint surface improved; the impact surface hardness was the largest and gradually decreased inward to the weld zone base metal hardness, with a small improvement in the tensile strength. Compared with the single treatment process, the microstructural and mechanical properties of the welded joint after A–DSUIT were comprehensively improved. The microhardness and tensile strength of the welded joint reached 200 HV and 615 MPa, respectively, for an increase of 45.8% and 61.8%, respectively. Observation of the fractures of the tensile specimens under the different treatment processes showed that the fractures before the aging treatment were mainly ductile fractures while those after were mainly brittle fractures. After DSUIT of the welded joints, a clear and dense plastic deformation layer was observed in the fracture of the tensile specimens and effectively improved the tensile properties of the welded joints. Under the EBSD characterization, the larger the residual compressive stress near the ultrasonic impact surface, the smaller the grain diameter and misorientation angle, and the lower the texture strength. Finally, after A–DSUIT, the hardness and tensile properties improved the most.

## 1. Introduction

As an aging heat treatment reinforced aluminum alloy, (Al–Zn–Mg–Cu) 7075 aluminum alloy exhibits high specific strength, good fracture toughness, and excellent low cycle fatigue resistance among other characteristics; it is widely used in the fields of transportation, aerospace, and aviation [1,2,3,4]. Tungsten inert gas welding, metal inert gas welding [5], plasma arc welding and laser welding are usually used for this alloy. Laser welding exhibits the advantages of a high energy density, fast welding speed, fine grain, high mechanical joint properties, and litter deformation, and achieving a narrow weld with this method is easy [6,7,8,9,10]. Therefore, this study uses laser welding to weld 7075 aluminum alloy.

In the welding process, 7075 aluminum alloy shows serious weldability problems, especially in the process of fusion welding, wherein the weld microstructure is loose, and the weld exhibits serious porosity and other defects [11], limiting the application of this alloy in actual production. To improve the comprehensive properties of 7075 aluminum alloy joints, post-weld treatment is an ideal and popular method [12,13,14]. Many post-weld treatment methods exist for welded joints, such as electron beam treatment [15,16], heat treatment and mechanical treatment. However, welded joints of 7075 aluminum alloy normally undergo post-weld heat and mechanical treatments, of which the heat treatment is usually aging treatment [17,18], and the mechanical treatment usually ultrasonic impact treatment (UIT) [19,20].

Safarbali et al. [21] studied the influence of post-weld heat treatment on dissimilar friction stir welding AA7075 and AA2024 joints, showing that the welded joints exhibit a higher microhardness and better mechanical properties after solution and aging treatment. Mohammad et al. [22] performed an aging treatment on AA6061 and AA2204 aluminum alloy friction stir welded joints and demonstrated that the strength and toughness of the welded joints after aging treatment increased by 92% and 96%, respectively. Liang Li et al. [23] discussed the strengthening mechanism of ultrasonic impact. Their results showed that UIT technology can significantly improve the microhardness, strength, and wear resistance of the material surface. Ultrasonic impact on the surface of a material can release the residual tensile stress, restore the beneficial compression stress, and improve the material properties. Li et al. [24] carried out UIT on high entropy alloy, showing that it exhibited no effect on the phase composition of a sample, but that the grain diameter of the sample surface after the treatment decreased and hardness of the sample surface increased significantly. Simultaneously, the surface roughness of the sample was reduced. However, a single post-weld treatment method induces limited improvements in the performance of welded joints and cannot comprehensively improve their microstructural and mechanical properties.

Although the aging treatment of welded joints alone improves the joints’ strength and microhardness, their surface defects and loose structures cannot be improved. This leads to a larger stress concentration on the surface of welded joints when a welded component is used, which is not conducive to a joint’s service life. Although UIT of welded joints alone can reduce surface defects and loose structures, the strength of joints after treatment is low, making the use of 7075 aluminum alloy welded joints unfavorable for high-strength operations. Therefore, to obtain a 7075 aluminum alloy laser-welded joint with a better overall performance, this study combines the effects of aging treatment on the strength and microhardness of welded joints with the effects of UIT on improving joint surface defects and loose structure. A welded joint compound treatment process, i.e., aging and double-sided UIT (A–DSUIT), is proposed. The purpose is to effectively improve the strength, microhardness, surface defects, and loose structure of the welded joint through a combination of aging treatment and UIT.

Here, we conduct post-weld aging treatment, post-weld DSUIT, and post-weld A–DSUIT for 7075 aluminum alloy laser-welded joints. The effect mechanism of A–DSUIT on the joints’ microstructural and mechanical properties is contrasted and analyzed. Hopefully, this study will lay a theoretical foundation for the application of 7075 aluminum alloy laser welding.

## 2. Materials and Methods

The test material was a 7075 aluminum alloy rolled sheet with a thickness of 3 mm (SW Aluminum, Chongqing, China); its chemical composition is shown in Table 1.

**Figure 1:**
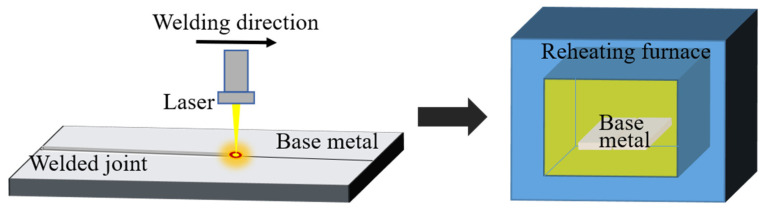
Schematic of laser welding and heat treatment test.

The welding equipment was an IPG YLS-10000 fiber laser (IPG Potonics Corporation, Santa Clara, CA, USA) and a KUKA welding robot (KUKA Roboter GmbH, Augsburg, Bavaria, Germany) was used to conduct butt welding to the 16 × 8 × 3 mm^3^ base metal. Moreover, T6 heat treatment was adopted for the post-weld heat treatment; the equipment used was a box-type resistance furnace, and the test accuracy was ±3 °C. The laser welding and heat treatment process parameters are shown in Table 2. The principles of the laser welding and heat treatment test are shown in Figure 1.

**Figure 2:**
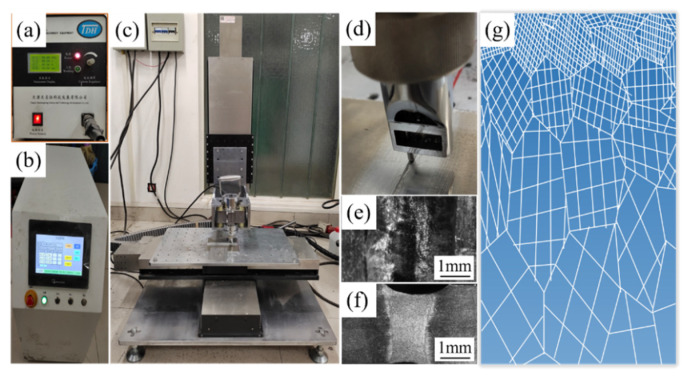
Schematic diagram of DSUIT. (**a**) Ultrasonic impact machine; (**b**) 3D sliding platform controller; (**c**) the composite system of the ultrasonic impact gun head and 3D slide table; (**d**) the working diagram of the welded joint treatment by ultrasonic impact needle; (**e**) the welded joint surface morphology after DSUIT; (**f**) the welded joint cross-section after ultrasonic impact; and (**g**) the schematic of the grain gradient refinement of the welded joint after DSUIT.

In the ultrasonic impact test, a UIT-125 ultrasonic impact machine (Tianjin Dongheng Science and Technology Development Co., Ltd., Tianjin, China) and a self-developed three-dimensional (3D) sliding platform system were combined to treat the welded joints. The DSUIT schematic is shown in Figure 2. The test impacted both the upper and lower surfaces of the welded joints; the movement of the impact needle from the beginning to the end of the welded joint was counted as one impact. The DSUIT test parameters are shown in Table 3.

**Figure 3:**
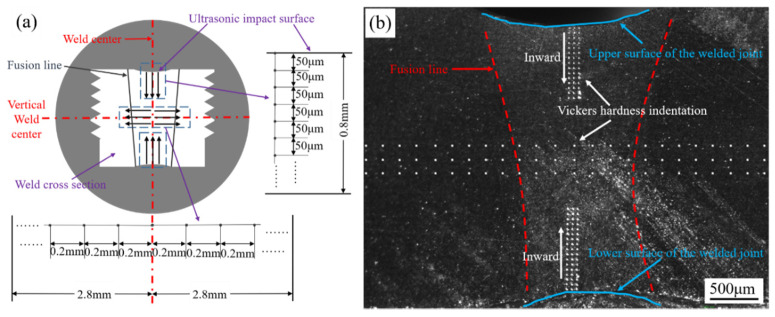
Vickers hardness test. (**a**) Test principle diagram and (**b**) schematic diagram of points collected for microhardness tests.

An optical microscope (Zeiss, Oberkochen, Jena, Germany) was used to observe the changes in the microstructure of the weld zone under different processes. An FEI-QUANTA 650 scanning electron microscope (FEI, Hillsborough, FL, USA.) was used to carry out the electron backscatter diffraction (EBSD) analysis of the welded joints under the different treatment processes, as well as a scanning electron microscopy (SEM) analysis of the fractures of the tensile specimens. Vickers hardness test (Matsuzawa, Akita, Japan) was used to test the microhardness of the welded joints under the different processes. The test loading force was 100 gf, the loading time was 10 s, and the average value was obtained by performing the test three times to reduce errors. The microhardness test method is shown in Figure 3 (due to the volume of the indenter itself, the first point in the longitudinal hardness test was 10 μm away from the impact surface). The tensile properties of the welded structural parts were tested using an SHT-4605 microcomputer-controlled electrohydraulic servo universal testing machine (Shanghai SanSi Metering Instrument Manufacturing Co., Ltd., Shanghai, China). The samples for each treatment were tested three times.

## 3. Results and Discussion

### 3.1. Effect of Aging Treatment on the Microstructure of the Welded Goints

**Figure 4:**
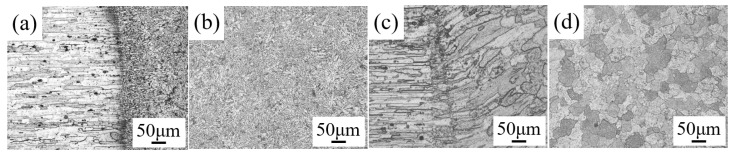
Microstructure of a welded joint before and after aging. (**a**) Microstructure near the fusion line before aging; (**b**) microstructure of the weld center before aging; (**c**) microstructure near the fusion line after aging; and (**d**) microstructure of the weld center after aging.

Figure 4 shows the changes in the microstructure of a 7075 aluminum alloy fiber laser-welded joint before and after aging. As shown in Figure 4a, the fusion line of the joint was very narrow, and the growth pattern of the fusion line toward the central grain of the weld zone was small columnar grain. It can be observed from Figure 4b that the microstructure of the weld zone of the alloy exhibited the characteristics of an as-cast microstructure, showing a dendritic network structure. The black spots in the figure are non-equilibrium eutectic phase α(Al)+T(AlZnMgCu). Based on the microstructure in Figure 4c, the fusion zone of the joint became wider after the aging and the columnar crystals near the fusion line grew upward. The microstructure in Figure 4d shows that after the treatment, the dendritic reticulated grains in the weld zone grew into an equiaxed grain structure and that most of the non-equilibrium eutectic phases were eliminated.

After the aging treatment, the dendrite structure of the 7075 aluminum alloy laser-welded joint was basically eliminated and transformed into an equiaxed crystal. This was because the solution treatment before the aging treatment recrystallized the dendritic structure of the welded joint and the dendrite segregation homogenized in the process of the solution heat treatment. Simultaneously, the non-equilibrium eutectic phase in the welded joint was eliminated, and the grains in the welded joint again nucleated and grew. The aging treatment following immediately caused the recrystallized grains to continue to grow. After the 24-h aging treatment, the grain structure completely grew from a dendritic structure to an equiaxed structure. The continuous treatment created continuous growth conditions for the fusion zone grains of the welded joint, which made the fusion zone wider and effectively improved the joint’s connection properties. In the aging process, the grain growth also improved the microhardness and strength of the welded joint, which effectively improved the joint’s mechanical properties. 

### 3.2. Effect of DSUIT on the Microstructure of the Welded Joint

**Figure 5:**
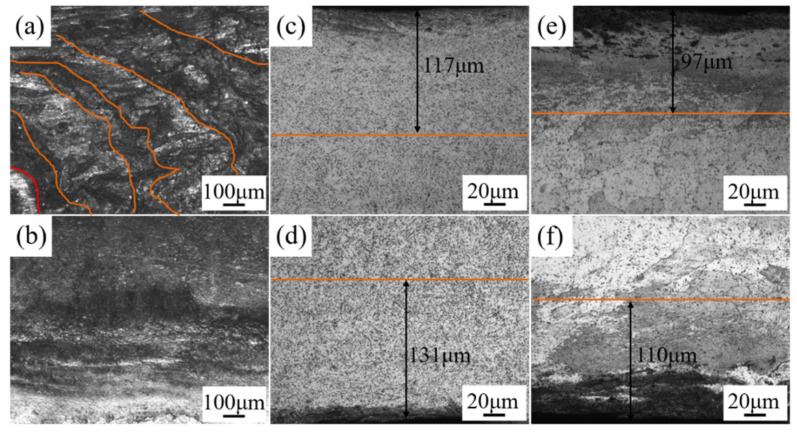
Microstructure of a welded joint after DSUIT. (**a**) As-welded (AW) joint surface morphology; (**b**) welded joint surface morphology of DSUIT; (**c**) plastic deformation layer (PDL) of the upper surface of welded joint after DSUIT; (**d**) PDL of the lower surface of welded joint after DSUIT; (**e**) PDL of the upper surface of welded joint after A–DSUIT; and (**f**) PDL of the lower surface of welded joint after A–DSUIT.

Figure 5 shows the microstructure of the 7075 aluminum alloy laser-welded joint surface before and after DSUIT and the microstructure of the welded joint cross-section after DSUIT.

According to the analysis of the microstructure results in Figure 5, the surface of the AW 7075 aluminum alloy joint demonstrated a regular peak structure that was very uneven. This would cause stress concentration at the joint during its use, which would not be conducive to its service life (Figure 5a). After the UIT on the welded joint, the surface of the weld zone became flat and smooth, and the welding surface defects were significantly reduced (Figure 5b). According to a comparative analysis of Figure 5c,d and Figure 5e,f), the thickness of the PDL on the lower surface of the welded joint after DSUIT was greater than that on the upper surface. This phenomenon was caused by the 7075 aluminum alloy welded joints of Zn and Mg elements at high-temperature volatilization and local depletion of these volatile elements (usually via irregular vaporization), leading to keyhole collapse so that the welded joint was lower than the base metal [25]. Therefore, during the UIT on the upper surface of the welded joint, the weld toe part was impacted first so that the ultrasonic impact strength on the joint’s upper surface was lower than that on the lower surface; hence the thickness of the PDL on the upper surface was small. Moreover, the thickness of the PDL of the welded joint after A–DSUIT was smaller than that DSUIT due to the improvement of the joint microhardness after aging. No phase transformation occurred in the welded joint after DSUIT, which only refined the grain diameter on the joint’s surface [26].

### 3.3. EBDS Analysis of Welded Joints under Different Processes

**Figure 6:**
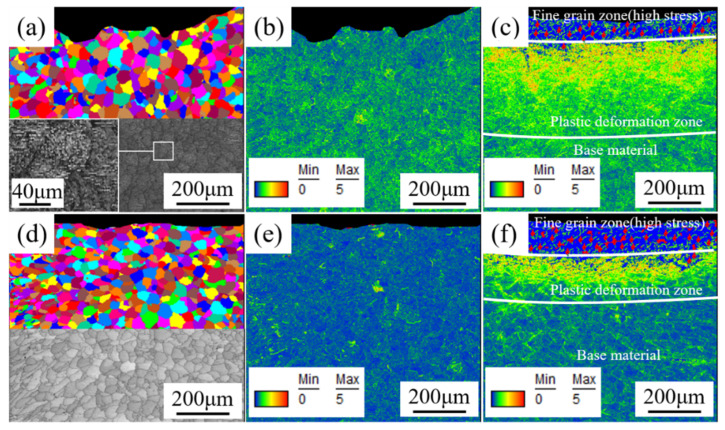
EBSD grain and stress distribution diagrams of the weld zone. (**a**) EBSD grain diagram of weld zone of the AW; (**b**) EBSD stress distribution diagram of weld zone of the AW; (**c**) EBSD stress distribution diagram of weld zone after DSUIT; (**d**) EBSD grain diagram of weld zone after aging; (**e**) EBSD stress distribution diagram of weld zone after aging; and (**f**) EBSD stress distribution diagram of weld zone after A–DSUIT.

**Figure 7:**
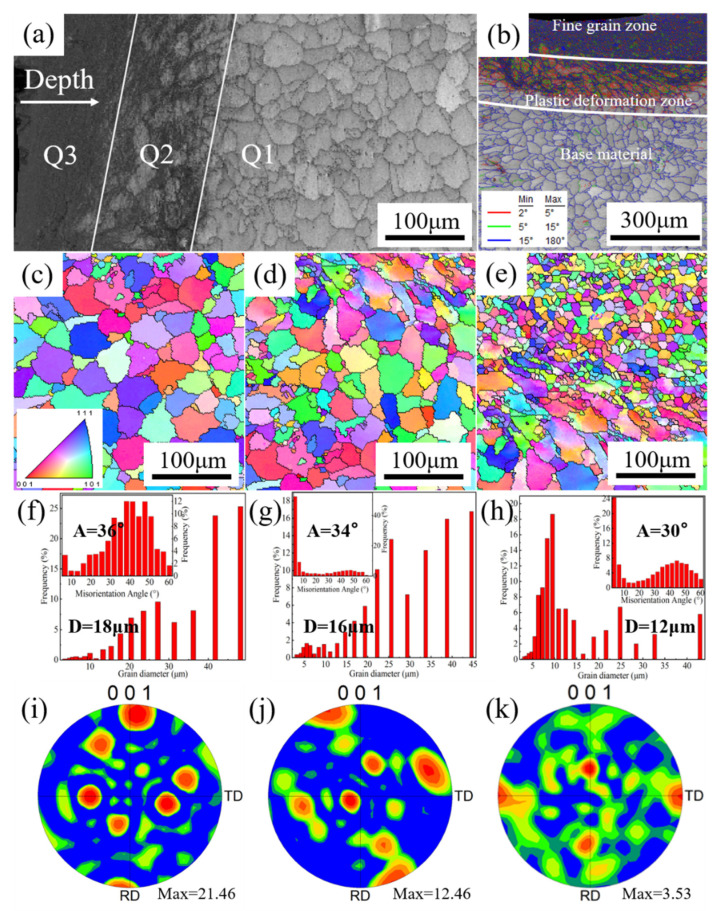
EBSD characterization. (**a**) Microstructure zones of weld zone after A–DSUIT; (**b**) misorientation angle distribution; (**c**–**e**) Q1–Q3 inverse pole figure; (**f**–**h**) Q1–Q3 average grain diameter and misorientation angle statistics; and (**i**–**k**) Q1–Q3 polar Figure.

To study the microstructure and stress changes of welded joints under the different treatment processes, the EBSD method was used to test the microstructure of the welded zone. TSL OIM Analysis software was used to analyze the EBSD test results, and the grain, stress distribution, misorientation angle distribution, inverse pole and pole figure were obtained. Moreover, the distribution of the grain diameter and misorientation angle in the weld zone was processed using TSL OIM Analysis, and the average grain diameter and average misorientation angle were calculated. The analysis results are shown in Figure 6 and Figure 7.

Figure 6 shows the EBSD grain and stress distribution diagrams of the weld zone. Comparing Figure 6a,b, researchers found that after the aging treatment of the welded joints, the grain diameter of the weld zone became uniform, and the zone’s grain structure changed from the original dendritic structure to an equiaxed structure. Moreover, comparing Figure 6b,e, it was clear that the stress at the welded joint was effectively eliminated after the aging treatment (the original stress of the AW joint was actually the residual tensile stress, which was not conducive to its service life). It can be seen from Figure 6c,f) that certain stress was introduced into the fine grain and plastic deformation zones of the welded joint after DSUIT (the stress introduced here was residual compressive stress, which was beneficial to the service life of the welded component [27]). The fine grain zone displayed red (high) stress (i.e., residual compressive stress), and the residual compressive stress near the welded joint surface was too large, which led to the missing phenomenon in the EBSD analysis process. Meanwhile, it was observed in Figure 6c,f that, because the hardness of the AW joint was lower than that of the aged joint, the PDL thickness of the former was larger than that of the latter, and the residual compressive stress introduced was deeper. However, the zone (base material) of the AW joint not affected by DSUIT showed greater residual tensile stress than that of the aged joint not affected by DSUIT, and the thickness of the stress layer introduced by DSUIT was very different from the overall thickness of the welded joint. Therefore, welded joints treated with A–DSUIT could show better properties in practical application.

In view of the microstructure changes in the plastic deformation zone, the A–DSUIT sample was selected as the research object, and the surface layer was further divided into three zones (Q1, Q2, and Q3) according to the grain morphology (Figure 7a). The grain morphology, grain diameter, misorientation angle, and texture strength of the three zones were characterized by EBSD analysis. After the weld zone was ultrasonically impacted, the grains in the near-surface zone were extruded and broken, and a large number of small-angle grain boundaries were formed in the plastic deformation zone of the weld zone (Figure 7b). Figure 7c shows the inverse pole figure of zone Q1; it can be seen that each grain exhibited its own independent orientation information with an equiaxed grain structure. The average grain diameter in Q1 was 18 μm, the and average misorientation angle was 36° (Figure 7f). According to the polar figure of Q1 in Figure 7i, the texture strength of this region was higher at plane <001>, and the texture strength was 21.46.

Figure 7d is the inverse pole figure of the microstructure of Q2, which was affected by the ultrasonic impact and produced a certain strain between Q1 and Q3. The columnar crystals in the microstructure were deformed and accompanied by the phenomenon of columnar crystal breaking. Compared with the columnar grain diameter and misorientation angle in Q1, the shape and size of the subsurface grains in Q2 changed, and the grain diameter and misorientation angle decreased, with an average grain diameter of 16 μm and average misorientation angle of 34° (Figure 7g). According to the polar figure of Q2 in Figure 7j, the texture strength of the subsurface zone at plane <001> was lower than that in Q1, and the texture strength was 12.46. Moreover, the texture orientation began to shift under the influence of the ultrasonic impact.

Figure 7e is the inverse pole figure of Q3, where severe plastic deformations occurred near the weld zone surface. The deformed columnar grains in this zone were broken into finer grains; the average misorientation angle in this zone decreased to 30°, while the average grain diameter decreased to 12 μm (Figure 7h. As can be seen from the polar figure of Q3 in Figure 7k, the texture peak of the strong plastic deformation zone on the weld zone surface decreased at plane <001>, and the texture strength decreased to 3.53. The decrease of the texture peak and strength was due to the large grain structure being broken by the impact during the DSUIT of the welded joint, which broke the original texture orientation and reduced the texture strength.

To sum up, proper aging treatment of the welded joint obviously eliminated the residual tensile stress inside the joint and made the grain size at the joint uniform. The DSUIT introduced a certain residual compressive stress to the welded joint, refined the grain size of the joint surface, and reduced the misorientation angle and texture strength of part of the weld zone. When the grain on the joint surface is refined, the surface defects were also reduced, and the texture in the plastic deformation zone was broken and refined. This reduced the joint’s texture strength, which was beneficial to the tensile property of the welded structural parts. As per the analysis, the two treatments exhibited obvious effects on improving the structure and mechanical properties of the welded joints, but the composite treatment was the best. Therefore, it would be feasible and effective to adopt the A–DSUIT process to improve the comprehensive properties of 7075 aluminum alloy laser-welded components, providing a theoretical basis for the laser welding of such components in practical applications.

### 3.4. Microhardness Analysis of the Welded Joints under Different Treatment Processes

**Figure 8:**
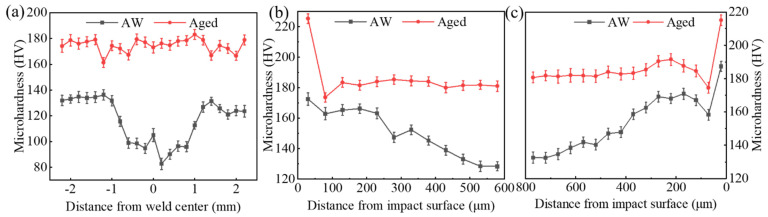
Vickers hardness. (**a**) Base metal–weld–base metal; (**b**) the upper surface of the welded joint is inward; and (**c**) the lower surface of the welded joint is inward.

The Vickers hardness of the welded joints under the different treatment processes was tested. Figure 8a shows the Vickers hardness curves of the vertical weld center from base metal–weld–base metal on the cross-section of the AW and aged joints. Figure 8b,c displays the inward Vickers hardness curves of the upper and lower surfaces of the weld zone after DSUIT of the AW and aged joints.

According to the Vickers hardness curve in Figure 8, the microhardness of the weld zone of the AW joint was lower than that of the base metal. The microhardness at the weld center was the lowest, about 100 HV; the microhardness in the heat-affected zone of the welded joint was the highest, about 140 HV; and the overall average microhardness of the welded joint was about 120 HV. When the welding members were aged, the overall average microhardness was stable around 175 HV, and the microhardness improvement rate of the welded joint was 45.8% (Figure 8a). As shown in Figure 8b,c, based on the microhardness data of the AW joint, DSUIT effectively improved the welded joint surface microhardness, which gradually decreased inward, and the maximum Vickers hardness of the surface affected by ultrasonic impact reached 180 HV. After aging, the welded joint hardness was relatively high, and the DSUIT only exhibited a certain effect on the hardness of the surface layer of fewer than 100 μm. After the ultrasonic impact of the aging welded joint, the Vickers hardness of the impact surface reached 200 HV. Therefore, the A–DSUIT process significantly improved the microhardness of the welded joint.

### 3.5. Tensile Properties and Fracture Morphology Analysis under the Different Processes

**Figure 9:**
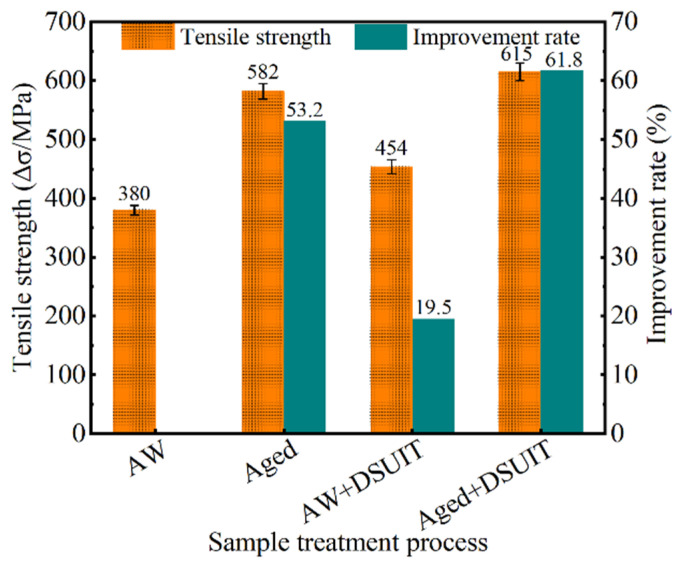
Tensile test results.

A tensile test was carried out on welded components treated by the different processes, and the test results are shown in Figure 9. The tensile strength of the AW joint was 380 MPa, and the joint strength after aging treatment, DSUIT, and A–DSUIT was 582, 454, and 615 MPa, respectively. The improvement rate of the welded joint strength was 29.7%, 19.5%, and 61.8%, respectively. The tensile strength of the welded joints treated by the three different processes was improved, among which A–DSUIT exhibited the most obvious effect. Therefore, the process of A–DSUIT could play an important role in improving the tensile properties of welded components.

**Figure 10:**
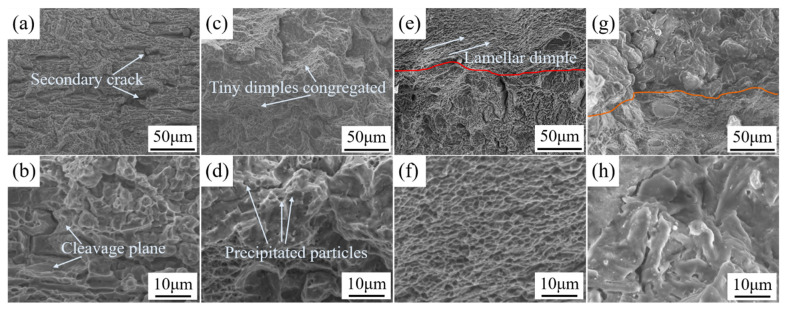
SEM of tensile fractures. (**a**) AW tensile fracture morphology (500×); (**b**) AW tensile fracture morphology (2000×); (**c**) aged tensile fracture morphology (500×); (**d**) aged tensile fracture morphology (2000×); (**e**) DSUIT tensile fracture morphology (500×); (**f**) DSUIT tensile fracture morphology (2000×); (**g**) A–DSUIT tensile fracture morphology (500×); and (**h**) A–DSUIT tensile fracture morphology (2000×).

The tensile fractures were observed by SEM, as shown in Figure 10, and researchers found that secondary cracks were generated on the fractured surface of the joints under the different treatment processes. As seen in Figure 10a,b, the tensile fracture morphology of the AW joint was mainly a dimple structure, followed by a partial cleavage plane observed from the fractured surface. As in Figure 10c,d, after the aging treatment, the surfaces fractures mainly comprised cleavage fractures, appearing as a congregation of tiny dimples (due to intensive Guinier Preston zones generated after the aging), and the fractured surface exhibited some precipitated particles (due to the accumulation of impurity elements during the aging process) (Figure 10d). The observation and analysis of the joint fracture morphology DSUIT and A–DSUIT showed that the morphology of the subsurface zone of the welded joints after ultrasonic impact was significantly different (Figure 10e,g). Researchers found that the impact zone of the AW fractured surface presented a regular fine lamellar dimple structure (Figure 10f), while the impact zone of the aged fractured surface presented a large cleavage fracture structure (Figure 10h).

Therefore, the fractures of the 7075 aluminum alloy laser-welded joints in the four states were ductile and brittle mixed fracture forms. The joints before the aging treatment had mainly ductile fractures, and the joints after the aging treatment had mainly brittle fractures. According to the tensile test results in Figure 10, the plastic deformation zone formed by DSUIT on the weld zone surface effectively improved the strength of the welded joints, improved the welding defects on the joint surface, and improved the microstructure and microhardness surface properties. Therefore, A–DSUIT exhibited an obvious effect on the improvement of the comprehensive mechanical properties of the 7075 aluminum alloy-laser welded joints.

## 4. Conclusions

In this study, the microstructural and mechanical properties of 7075 aluminum alloy laser-welded joints under aging treatment, DSUIT, and A–DSUIT were compared and analyzed. The conclusions were drawn as follows:

(1) After the aging treatment, the weld zone of the 7075 aluminum alloy laser-welded joints changed from a dendritic to an equiaxed grain structure, and the grain became uniform. The non-equilibrium eutectic phase in the weld zone was eliminated after this treatment.

(2) DSUIT produced a certain PLD thickness in the weld zone. The average thickness of the PDL on the upper and lower surfaces of the welded joint in the welded state was about 124 μm, and the average thickness of the PDL on the upper and lower surfaces of the weld in the aging state is about 103 μm. After the DSUIT, the welding defects on the joint surface were reduced.

(3) The aging treatment eliminated the residual tensile stress of the welded joint, and DSUIT introduced a certain residual compressive stress to the joint. The residual compressive stress near the surface of the weld zone was the largest.

(4) After DSUIT, the welded zone was divided into three zones: fine grain zone, plastic deformation zone, and base metal zone. The grain diameter and misorientation angle from the base metal to the fine grain zone decreased gradually, as did the texture strength.

(5) The aging treatment effectively improved the shortcoming of low hardness in the welded joint. After the treatment, the Vickers hardness of the joint was about 180 HV, while that of the joint surface with A–DSUIT reached up to 220 HV.

(6) The tensile fractures in the four states were all ductile/brittle mixed fractures. The joint treated by A–DSUIT demonstrated the highest tensile strength at 615 MPa, which was 61.8% higher than that of the AW. After ultrasonic impact, the fracture surface of the joint exhibited an obvious boundary. The boundary of the joint treated DSUIT manifested as a dense dimple structure. The boundary of the joint treated by A–DSUIT manifested as a fractured structure of intergranular cleavage.

## Figures and Tables

**Table 1:** Chemical composition of 7075 aluminum alloy (mass, fraction, %).

Element	Mg	Zn	Cu	Cr	Fe	Mn	Ti	Si	Al
GB/T3190	2.1–2.9	5.1–6.1	1.2–2.0	0.18–0.28	≤0.5	≤0.3	≤0.2	≤0.4	balance
Reinspection	2.23	5.54	1.41	0.19	0.43	0.22	0.14	0.31	balance

**Table 2:** Laser welding and heat treatment process parameters.

Laser Welding Parameters	Power/W	Welding Speed *v*/m·s^−1^	Defocusing Distance/mm	Upper/Lower Shielding Flow *L*/min	T6 Heat Treatment Process Parameters	Solution Temperature × Solution Time /°C × h	Quench	Aging Temperature × Aging Time/°C × h
	2700	0.054	+4	15/15		470 × 2	Water	120 × 24

**Table 3:** DSUIT test parameters.

Working Frequency *f*/KHz	Working Current *I*/A	Diameter of Impact Needle *d*/mm	Velocity of Impact Needle Movement *v*/mm·s^−1^	Impact Times */N*
25	2.0	3	30	150

## Data Availability

The data presented in this study are available on request from the corresponding author.
